# Supplementary material for: A Likelihood Approach for Real-Time Calibration of Stochastic Compartmental Epidemic Models
Source: PLoS Comput Biol. 2017 Jan 17;13(1):e1005257. doi: 10.1371/journal.pcbi.1005257 (PMC5240920; doi:10.1371/journal.pcbi.1005257)
Supplement: S1 File — (TAR.GZ) [file pcbi.1005257.s014.tar.gz › HSPH_Online-SI-Revision/Additions/EnKF08/n100-extreme-SEITR/n100-extreme-SEITR_EnKF_table8.pdf]

1	R0	{1.38654, 9.85565}	{2.73841, 6.51066}	{{2.27337, 3.7775}, {3., 8.82951}}	{97.4997, 35.5953}	inf	78.	4125.49	{1068.93, 14 737.1}	532.26	0.640952	newly	382.	5827.91	{1501., 18 055.}	1429.6	0.893267	Reff	0.108483	0.925749	{0.469066, 2.27334}	759.164	ar	92 379.	185 895.	{162 585., 248 826.}	101.231
2	R0	{2.36629, 12.2029}	{2.15992, 7.86314}	{{1.28413, 2.96309}, {3., 11.019}}	{35.9667, 37.0179}	inf	5.	5154.95	{1919.49, 13 953.6}	650.251	0.70671	newly	300.	7263.33	{923., 17 440.}	2323.06	0.957265	Reff	0.0235682	0.387996	{0.0378295, 1.2829}	1549.52	ar	99 022.	187 796.	{173 177., 239 633.}	89.6504
3	R0	{2.32253, 12.836}	{2.54231, 4.6396}	{{1.763, 3.28941}, {3., 6.1965}}	{27.2907, 63.8548}	inf	20.	1478.47	{120.458, 1109.15}	74.6819	0.230883	newly	463.	1849.65	{142., 1793.}	322.339	0.458277	Reff	0.0245491	0.477171	{0.121781, 0.608319}	1844.06	ar	98 997.	164 976.	{154 648., 171 287.}	66.6477
4	R0	{1.79075, 11.2058}	{2.53254, 8.93291}	{{1.82772, 2.95313}, {3., 12.6933}}	{41.4237, 35.2767}	inf	31.	10 078.8	{5130.68, 7816.73}	1119.93	0.928897	newly	412.	9714.94	{2861., 11 414.}	2259.72	1.09821	Reff	0.0574114	0.688646	{0.112821, 1.82739}	1103.07	ar	96 920.	208 694.	{186 746., 264 415.}	115.326
5	R0	{2.26952, 9.62371}	{2.51224, 1.92937}	{{1.83867, 0.181269}, {3., 6.34523}}	{26.6416, 79.9519}	inf	0.	691.746	{257.567, 1914.17}	271.126	0.361958	newly	78.	6494.89	{313., 6624.}	8227.42	1.16806	Reff	0.0257137	0.605404	{0.195968, 1.36054}	2254.41	ar	98 881.	183 051.	{169 461., 246 931.}	85.123
6	R0	{1.57191, 6.89004}	{2.17311, 7.91975}	{{0.982755, 4.57523}, {3., 10.9914}}	{68.9573, 38.9538}	inf	5.	4631.34	{1353.06, 9214.88}	8476.56	1.74069	newly	40.	4366.85	{1167., 13 004.}	10 817.1	1.74313	Reff	0.077951	0.477001	{0.105199, 0.979431}	516.428	ar	95 046.	174 286.	{164 399., 204 005.}	83.3707
7	R0	{1.99226, 17.2339}	{1.844, 12.9771}	{{0.0294457, 6.81482}, {3., 17.6813}}	{69.1525, 26.7353}	inf	78.	6567.87	{1098.92, 20 978.8}	177.882	0.321455	newly	982.	4316.75	{503., 9128.}	383.327	0.406687	Reff	0.0456427	0.22872	{0.00679977, 0.459324}	448.15	ar	97 992.	163 709.	{151 813., 196 598.}	67.0641
8	R0	{2.63369, 15.1879}	{2.22562, 8.54633}	{{0.719303, 2.94597}, {3., 11.1158}}	{33.8538, 43.7294}	inf	5.	6245.02	{298.542, 5251.66}	360.905	0.600494	newly	510.	4492.44	{151., 7815.}	800.318	0.829772	Reff	0.0154861	0.474401	{0.14132, 0.718895}	2965.14	ar	99 451.	184 342.	{172 356., 189 641.}	85.3601
9	R0	{2.53271, 9.90552}	{2.52394, 6.16437}	{{1.8097, 3.3059}, {3., 7.71906}}	{22.4867, 37.7684}	inf	2.	8366.95	{1319.69, 6081.26}	4795.67	1.36644	newly	96.	7581.13	{1323., 33 995.}	7798.78	1.55619	Reff	0.0167159	0.504411	{0.234578, 0.779704}	2919.19	ar	99 355.	187 814.	{172 111., 235 183.}	89.0334
10	R0	{2.48791, 18.9003}	{2.48245, 6.00786}	{{1.89883, 0.458042}, {3., 13.057}}	{22.0377, 68.2129}	inf	42.	4146.23	{1454., 5659.11}	52.1687	0.223641	newly	1167.	21 527.5	{763., 80 827.}	1757.01	0.824404	Reff	0.0212716	0.997696	{0.265705, 1.89881}	4591.8	ar	99 291.	214 220.	{185 492., 269 489.}	115.749
11	R0	{2.84414, 18.4765}	{2.64747, 13.3967}	{{1.98678, 9.14201}, {3., 17.7596}}	{14.2581, 27.6323}	inf	13.	11 001.9	{670.265, 22 209.9}	295.24	0.624224	newly	1003.	5917.96	{259., 13 918.}	528.873	0.736364	Reff	0.0117463	0.726793	{0.120348, 1.9871}	6087.42	ar	99 654.	194 531.	{160 976., 273 597.}	95.2068
12	R0	{2.29369, 14.622}	{2.66022, 10.6095}	{{2.00064, 4.28933}, {3., 18.4772}}	{24.6677, 45.1198}	inf	14.	4020.88	{332.214, 8751.33}	229.02	0.549136	newly	542.	3022.88	{300., 5811.}	473.786	0.614983	Reff	0.0247948	0.637722	{0.182456, 1.72426}	2477.07	ar	98 982.	183 260.	{168 392., 221 441.}	85.1452
13	R0	{2.73413, 16.7529}	{2.3641, 5.99868}	{{1.40827, 1.04585}, {3., 11.8256}}	{25.2027, 64.3618}	inf	10.	13 835.4	{2044.62, 29 372.5}	587.205	0.671553	newly	706.	12 999.9	{1596., 20 944.}	1748.02	1.07013	Reff	0.0138347	0.590964	{0.065538, 0.962408}	4171.61	ar	99 543.	211 803.	{183 128., 270 231.}	112.775
14	R0	{1.79554, 16.5943}	{2.57221, 5.24237}	{{1.95661, 0.709261}, {3., 9.19512}}	{43.2556, 68.4086}	inf	185.	2414.69	{145.511, 1693.68}	94.8392	0.657258	newly	1313.	3377.24	{132., 9064.}	217.898	0.532748	Reff	0.0662734	0.508922	{0.0382385, 1.68913}	685.438	ar	96 821.	168 374.	{151 111., 232 652.}	73.9019
15	R0	{1.91429, 13.7771}	{2.73609, 10.472}	{{2.28674, 5.84188}, {3., 14.5405}}	{42.93, 28.8691}	inf	37.	8489.93	{275.573, 17 038.4}	508.472	0.632758	newly	621.	7580.79	{118., 24 312.}	1151.63	0.814295	Reff	0.0501544	0.685385	{0.066565, 2.28672}	1270.36	ar	97 541.	191 486.	{162 645., 287 344.}	96.3136
16	R0	{1.42094, 10.5871}	{2.28874, 9.77035}	{{1.34487, 3.93967}, {3., 14.8384}}	{63.9613, 40.7945}	inf	126.	5349.88	{542.775, 4157.25}	363.888	0.530012	newly	644.	4056.08	{227., 4680.}	552.742	0.638697	Reff	0.103388	0.422582	{0.17474, 0.703694}	310.685	ar	93 044.	168 407.	{157 081., 184 817.}	80.9967
17	R0	{1.45515, 6.16466}	{2.4765, 4.33741}	{{1.86195, 2.43462}, {3., 7.90417}}	{70.1885, 48.6822}	inf	5.	4136.68	{1780.73, 1780.73}	8173.35	1.62747	newly	41.	5122.73	{1132., 10 685.}	12 394.5	1.7696	Reff	0.0982954	0.477894	{0.0419089, 1.57259}	411.299	ar	93 254.	189 313.	{173 401., 234 190.}	103.008
18	R0	{2.85669, 13.2416}	{2.00431, 8.23354}	{{0.914221, 3.92529}, {3., 11.7322}}	{34.6541, 37.8207}	inf	1.	4807.33	{372.668, 12 591.4}	538.567	0.572193	newly	314.	4635.91	{339., 12 074.}	1386.42	0.742396	Reff	0.0106269	0.515864	{0.157564, 0.914273}	4755.71	ar	99 644.	181 431.	{166 860., 214 629.}	82.0795
19	R0	{2.56417, 17.9558}	{2.35765, 5.48423}	{{0.679809, 0.896205}, {3., 7.60914}}	{29.81, 69.457}	inf	35.	9581.79	{103.587, 34 886.4}	229.945	0.547573	newly	1136.	12 654.7	{92., 49 953.}	1043.15	0.851879	Reff	0.01818	0.482685	{0.161872, 0.69504}	2555.04	ar	99 389.	170 956.	{145 742., 259 072.}	72.0074
20	R0	{2.27517, 11.1864}	{1.98492, 15.942}	{{0.882099, 9.876}, {3., 18.4876}}	{45.8897, 49.072}	inf	9.	6975.95	{577.968, 17 641.}	1418.25	0.859119	newly	219.	3061.11	{206., 6655.}	1302.07	0.811871	Reff	0.0255502	0.629581	{0.160333, 0.927154}	2364.82	ar	98 894.	183 329.	{167 130., 222 174.}	85.3788
21	R0	{2.35735, 10.4735}	{2.47447, 2.21559}	{{1.80528, 0.326683}, {3., 6.46752}}	{25.5647, 78.8458}	inf	3.	808.725	{640.06, 1255.9}	171.953	0.399235	newly	156.	1722.16	{653., 1638.}	1005.22	0.831636	Reff	0.0230549	0.423545	{0.201593, 0.648054}	1740.85	ar	99 036.	170 091.	{163 932., 180 489.}	71.747
22	R0	{1.79551, 11.9179}	{2.67389, 3.83795}	{{2.16381, 3.18987}, {3., 4.01576}}	{48.9207, 67.7967}	inf	31.	2822.16	{562.148, 4422.73}	240.649	0.315393	newly	400.	5112.99	{636., 13 090.}	1179.69	0.6815	Reff	0.0555172	0.663371	{0.113702, 1.87309}	1094.89	ar	97 033.	204 473.	{181 887., 263 007.}	110.725
23	R0	{1.23545, 6.31687}	{1.94708, 3.78194}	{{0.230858, 1.81246}, {3., 4.65928}}	{119.153, 40.1295}	inf	19.	9018.58	{647.225, 14 431.1}	9009.68	1.68286	newly	79.	9909.79	{944., 17 914.}	12 444.	1.83534	Reff	0.139655	0.426975	{0.0526794, 0.796808}	246.73	ar	88 739.	207 445.	{187 871., 226 606.}	133.77
24	R0	{2.24089, 10.1789}	{2.06958, 8.34707}	{{0.78241, 5.25131}, {3., 11.7523}}	{46.9402, 33.726}	inf	7.	7879.87	{1691.37, 15 290.3}	2170.85	1.0502	newly	171.	5152.51	{814., 10 418.}	2913.16	1.20488	Reff	0.025703	0.469134	{0.13926, 0.660114}	1726.09	ar	98 868.	192 587.	{171 826., 255 384.}	94.7923
25	R0	{1.57298, 10.2607}	{2.46312, 13.5369}	{{1.82289, 9.22664}, {3., 16.6734}}	{56.5894, 37.2211}	inf	47.	5725.68	{775.768, 8215.5}	567.363	0.621009	newly	443.	3106.53	{285., 4676.}	616.503	0.612693	Reff	0.0819837	0.438547	{0.0363073, 0.608399}	457.758	ar	94 957.	166 710.	{151 274., 208 494.}	75.5642
26	R0	{1.2154, 8.2576}	{2.11002, 3.30575}	{{0.362431, 2.39615}, {3., 4.80524}}	{116.854, 60.7617}	inf	88.	3396.78	{1203.1, 3861.28}	553.598	0.606365	newly	345.	4293.87	{1155., 7375.}	1149.32	0.90745	Reff	0.143952	0.512299	{0.117687, 0.664502}	266.381	ar	88 421.	167 465.	{156 803., 191 994.}	89.3954
27	R0	{2.22841, 18.7983}	{1.99387, 10.9388}	{{0.0213633, 7.21702}, {3., 13.8591}}	{54.6973, 41.8094}	inf	67.	7668.53	{2791.51, 10 371.6}	134.025	0.245362	newly	1079.	4630.74	{1349., 10 991.}	333.355	0.434629	Reff	0.0322005	0.408063	{0.00319947, 0.711439}	1202.53	ar	98 756.	172 095.	{156 199., 220 010.}	74.2626
28	R0	{1.27778, 5.36767}	{2.48173, 4.09307}	{{1.84829, 3.59336}, {3., 4.4625}}	{94.2221, 23.7459}	inf	7.	875.95	{305.462, 776.24}	2478.17	1.2	newly	32.	1436.94	{263., 1315.}	4392.19	1.28506	Reff	0.130065	0.488442	{0.189124, 0.936593}	278.082	ar	89 843.	176 408.	{166 292., 192 290.}	96.3516
29	R0	{2.38042, 15.8901}	{2.65092, 5.69669}	{{2.09226, 2.05537}, {3., 9.32774}}	{20.5636, 64.1494}	inf	37.	2793.13	{431.082, 7710.98}	82.1796	0.346616	newly	849.	7099.59	{410., 17 693.}	763.099	0.533806	Reff	0.0237804	0.686441	{0.16071, 1.71452}	2786.58	ar	99 112.	192 058.	{174 233., 256 886.}	93.7784
30	R0	{2.53188, 12.7607}	{2.6263, 5.58625}	{{1.93291, 3.54316}, {3., 7.27287}}	{19.7971, 56.223}	inf	11.	5110.57	{459.921, 2642.58}	436.195	0.428403	newly	428.	5739.1	{317., 3623.}	1249.9	0.655124	Reff	0.0182295	0.509453	{0.246396, 0.732919}	2696.44	ar	99 317.	179 785.	{163 310., 197 619.}	81.0215
31	R0	{1.24482, 10.5471}	{2.14713, 7.5026}	{{0.054478, 3.74181}, {3., 11.5094}}	{127.733, 38.0658}	inf	170.	4723.81	{615.606, 5416.82}	285.049	0.520815	newly	689.	3428.71	{503., 5859.}	409.491	0.627875	Reff	0.142009	0.432431	{0.00779982, 0.775787}	256.473	ar	89 097.	188 572.	{177 508., 212 336.}	111.648
32	R0	{2.30446, 17.9607}	{2.56401, 11.317}	{{1.98607, 3.16086}, {3., 14.3168}}	{23.145, 36.9904}	inf	48.	5800.7	{391.708, 16 279.1}	139.275	0.516609	newly	1094.	3403.46	{212., 8775.}	259.943	0.54502	Reff	0.0266626	0.754607	{0.14034, 1.98605}	2730.21	ar	99 028.	185 427.	{161 485., 259 834.}	87.2475
33	R0	{2.31579, 11.8236}	{2.57801, 3.57791}	{{1.85947, 1.29872}, {3., 5.37902}}	{25.9043, 69.7393}	inf	5.	1796.68	{247.566, 1692.38}	290.802	0.313013	newly	197.	2635.27	{298., 4206.}	1238.46	0.693327	Reff	0.0234821	0.426174	{0.117072, 0.921156}	1714.89	ar	99 012.	167 233.	{154 728., 195 652.}	68.902
34	R0	{1.6678, 10.6744}	{2.59317, 2.59976}	{{1.6345, 1.7823}, {3., 2.71491}}	{57.422, 75.6449}	inf	36.	3542.57	{1840.57, 3485.29}	522.788	0.59084	newly	278.	7955.33	{1815., 9927.}	2761.68	1.08737	Reff	0.0712985	0.579198	{0.122884, 1.48654}	714.033	ar	95 809.	180 302.	{164 822., 211 462.}	88.1886
35	R0	{1.65476, 7.07559}	{2.5593, 4.63076}	{{1.84027, 2.39586}, {3., 7.80748}}	{54.663, 42.8774}	inf	5.	1905.92	{241.719, 961.438}	2671.48	1.08062	newly	50.	2205.32	{244., 3495.}	4319.64	1.29126	Reff	0.0705259	0.523757	{0.0384091, 1.10257}	660.226	ar	95 746.	172 308.	{160 268., 200 063.}	79.9635
36	R0	{2.71807, 18.5959}	{2.65267, 8.41522}	{{2.03518, 1.22597}, {3., 19.049}}	{15.683, 55.7764}	inf	24.	9411.79	{966.271, 10 340.3}	169.006	0.391413	newly	1211.	9904.58	{1681., 21 826.}	723.143	0.706078	Reff	0.0154386	0.720404	{0.253085, 2.03516}	4566.42	ar	99 540.	187 485.	{166 133., 251 202.}	88.351
37	R0	{2.6202, 12.7552}	{2.23856, 4.86808}	{{0.869681, 3.4824}, {3., 11.8295}}	{31.6697, 66.791}	inf	9.	4839.46	{2632.04, 8397.21}	455.024	0.593431	newly	383.	5198.93	{2025., 8254.}	1257.42	0.937035	Reff	0.016088	0.34537	{0.114225, 0.497392}	2046.75	ar	99 426.	200 361.	{184 175., 232 356.}	101.518
38	R0	{1.47672, 9.81651}	{2.23021, 8.75058}	{{0.763225, 3.48869}, {3., 15.6499}}	{82.7146, 45.3195}	inf	40.	9216.56	{2156.45, 17 751.1}	1521.94	1.04927	newly	323.	7759.34	{1502., 15 466.}	2303.34	1.17004	Reff	0.0934468	0.647229	{0.23273, 0.908425}	592.925	ar	93 815.	178 670.	{158 877., 206 759.}	90.4495
39	R0	{1.90004, 12.3313}	{2.59401, 9.83761}	{{1.97784, 3.3004}, {3., 13.4115}}	{36.5239, 25.1921}	inf	30.	7746.64	{1707.92, 13 389.1}	635.965	0.684061	newly	464.	5820.96	{967., 8432.}	1156.69	0.873696	Reff	0.0517191	0.8873	{0.476216, 1.97782}	1615.61	ar	97 413.	197 432.	{171 920., 267 027.}	102.675
40	R0	{2.64218, 18.5237}	{1.67219, 12.5947}	{{0.180292, 8.46563}, {3., 15.2173}}	{49.5788, 32.0079}	inf	25.	8967.22	{1472.22, 27 452.4}	244.411	0.347769	newly	874.	4672.21	{617., 16 748.}	449.326	0.492698	Reff	0.0162494	0.406026	{0.039577, 0.655062}	2400.35	ar	99 474.	188 516.	{172 344., 227 645.}	89.5131
41	R0	{1.6945, 7.17996}	{2.43204, 4.41824}	{{1.70919, 2.73237}, {3., 6.03951}}	{43.5257, 38.4642}	inf	4.	1105.38	{225.483, 3202.2}	973.182	0.824503	newly	74.	1446.93	{173., 2782.}	1855.31	0.993862	Reff	0.0626626	0.423161	{0.121144, 0.820773}	583.458	ar	96 332.	184 312.	{174 856., 197 016.}	91.3299
42	R0	{1.84559, 18.0074}	{2.36548, 6.60689}	{{1.40952, 2.66662}, {3., 15.3155}}	{46.8903, 63.3101}	inf	180.	5969.02	{692.654, 11 907.5}	106.974	0.34129	newly	1374.	9219.52	{319., 19 257.}	591.017	0.566603	Reff	0.0586713	0.61111	{0.228068, 1.4107}	943.191	ar	97 358.	172 618.	{153 350., 216 805.}	77.3022
43	R0	{2.24965, 17.0361}	{2.63479, 5.25104}	{{2.01296, 0.897416}, {3., 10.158}}	{24.9059, 69.177}	inf	59.	5682.06	{1724.42, 4998.82}	99.5252	0.244033	newly	1018.	11 741.3	{2097., 20 255.}	1056.86	0.702681	Reff	0.0319	0.574641	{0.232252, 0.662281}	1703.03	ar	98 770.	190 308.	{173 501., 257 969.}	92.6777
44	R0	{2.54324, 15.7967}	{2.47969, 4.12526}	{{1.99085, 2.6006}, {3., 4.48675}}	{20.0995, 74.9482}	inf	13.	1997.74	{751.367, 2485.98}	65.5276	0.188381	newly	626.	2561.97	{884., 5307.}	315.014	0.478604	Reff	0.0177772	0.477836	{0.197785, 0.672375}	2588.36	ar	99 354.	182 122.	{171 951., 205 850.}	83.3065
45	R0	{2.54943, 14.8032}	{2.34005, 7.81435}	{{1.62441, 3.07341}, {3., 12.5404}}	{24.8258, 47.2117}	inf	8.	7316.11	{3887.46, 12 925.2}	436.372	0.562337	newly	536.	10 033.6	{1797., 29 523.}	1771.93	0.9005	Reff	0.0170047	0.602334	{0.12877, 1.65518}	3442.16	ar	99 380.	194 540.	{172 905., 254 314.}	95.7541
46	R0	{2.37755, 12.6461}	{2.48058, 4.53936}	{{1.89486, 2.97547}, {3., 6.71903}}	{23.4176, 64.1047}	inf	9.	3884.32	{409.625, 3563.43}	354.543	0.3553	newly	377.	5242.21	{348., 6676.}	1300.56	0.730187	Reff	0.0224678	0.681105	{0.189864, 1.39576}	2931.47	ar	99 096.	178 942.	{159 507., 215 904.}	80.5741
47	R0	{1.69279, 11.6636}	{2.47841, 13.5542}	{{1.89955, 9.88452}, {3., 17.1137}}	{46.4098, 24.2545}	inf	61.	11 685.7	{3035.96, 20 062.9}	896.24	0.809491	newly	550.	5874.9	{1250., 9306.}	969.102	0.814159	Reff	0.0676608	0.671312	{0.160697, 1.74418}	896.572	ar	96 214.	196 019.	{166 791., 268 801.}	103.732
48	R0	{1.82402, 10.0038}	{2.36546, 8.43423}	{{1.38443, 3.08167}, {3., 10.4637}}	{48.9619, 18.618}	inf	27.	6655.57	{586.571, 14 094.2}	1260.62	1.03156	newly	248.	6426.46	{390., 8379.}	2501.16	1.18581	Reff	0.054447	0.533745	{0.114182, 0.998214}	881.143	ar	97 076.	208 871.	{188 418., 260 770.}	115.162
49	R0	{1.6424, 11.1667}	{2.68442, 7.43059}	{{1.87278, 1.99918}, {3., 18.3723}}	{63.4452, 67.0045}	inf	29.	10 829.7	{2126.55, 9911.51}	1513.52	0.989428	newly	321.	14 109.7	{1656., 29 298.}	4295.55	1.24838	Reff	0.0752383	0.517157	{0.0690231, 0.902638}	592.495	ar	95 546.	198 995.	{173 636., 237 181.}	108.272
50	R0	{2.05638, 14.3048}	{2.30458, 11.2503}	{{0.201578, 7.68806}, {3., 13.3705}}	{55.7895, 23.3015}	inf	38.	7314.04	{1114.86, 7190.84}	373.249	0.466394	newly	636.	4090.71	{461., 7136.}	554.882	0.560934	Reff	0.0397498	0.279129	{0.0275886, 0.607592}	609.013	ar	98 194.	175 995.	{165 134., 179 388.}	79.2314
